# Situation, Background, Assessment, Recommendation (SBAR) Education for Health Care Students: Assessment of a Training Program

**DOI:** 10.15766/mep_2374-8265.11293

**Published:** 2023-01-03

**Authors:** Beth P. Davis, Sally A. Mitchell, Jeannie Weston, Catherine Dragon, Munish Luthra, James Kim, Hugh Stoddard, Douglas Ander

**Affiliations:** 1 Associate Professor, Division of Physical Therapy, Department of Rehabilitation Medicine, Emory University School of Medicine; 2 Associate Professor of Clinical Anesthesia, Vice Chair of Education, and Statewide Assistant Clerkship Director, Department of Anesthesia, Indiana University School of Medicine; 3 Assistant Professor, Emory University Nell Hodgson Woodruff School of Nursing; 4 Assistant Professor, Physician Assistant Program, Department of Family and Preventive Medicine, Emory University School of Medicine; 5 Assistant Professor, Department of Medicine, Emory University School of Medicine; 6 Associate Professor, Department of Medicine, Emory University School of Medicine; 7 Professor, Department of Medicine, Emory University School of Medicine; 8 Professor, Department of Emergency Medicine, Emory University School of Medicine

**Keywords:** SBAR, Communication Skills, Interprofessional Education, Program Evaluation, Quality Improvement/Patient Safety

## Abstract

**Introduction:**

Interprofessional communication failures are estimated to be a factor in two-thirds of serious health care–related accidents. Using a standardized communication protocol during transfer of patient information between providers improves patient safety. An interprofessional education (IPE) event for first-year health professions students was designed using the Situation, Background, Assessment, Recommendation (SBAR) tool as a structured communication framework. IPE literature, including a valid measurement tool specifically tailored for SBAR, was utilized to design the Interprofessional Team Training Day (ITTD) and evaluate learner gains in SBAR skills.

**Methods:**

Learners from six educational programs participated in ITTD, which consisted of didactics, small-group discussion, and role-play using the SBAR protocol. Individual learners were assessed using the SBAR Brief Assessment Rubric for Learner Assessment (SBAR-LA) on SBAR communication skills before and after the ITTD event. Learners received a written clinical vignette and submitted video recordings of themselves simulating the use of SBAR to communicate to another health care professional. Pre- and postrecordings were scored using the SBAR-LA rubric. Normalized gain scores were calculated to estimate the improvement attributable to ITTD.

**Results:**

SBAR-LA scores increased for 60% of participants. For skills not demonstrated before the event, the average learner acquired 44% of those skills from ITTD. Learners demonstrated statistically significant increases for five of 10 SBAR-LA skills.

**Discussion:**

The value to patient safety of utilizing structured communication between health care providers is proven; however, evaluating IPE teaching of communication skills effectiveness is challenging. Using SBAR-LA, communication skills were shown to improve following ITTD.

## Educational Objectives

By the end of this session, learners will be able to:

1. Describe to other health care professionals their own role and responsibilities as related to the collaborative care of a patient in the provided scenario (Interprofessional Education Collaborative [IPEC] subcompetencies RR1 and RR2).
2. Communicate with team members to identify each member's unique abilities and responsibilities as related to the collaborative care of a patient in the provided scenario (IPEC subcompetencies RR6, CC4, and CC7).
3. Reflectively explain the unique and complementary roles and responsibilities of at least two other health care professionals and how the team works together as related to collaborative care of a patient in the provided scenario (IPEC subcompetencies VE4, VE5, and RR4).
4. Discuss among team members the importance of interprofessional teamwork for improving patient care and outcomes for the patient in the provided scenario (IPEC subcompetencies CC8, RR10, and VE1).
5. Effectively communicate to another health care professional about a provided/simulated patient's condition using the Situation, Background, Assessment, Recommendation situational briefing tool (IPEC subcompetencies CC1, CC2, and CC3).

## Introduction

The 1999 Institute of Medicine report *To Err Is Human* sparked national attention and a prioritization of health care quality and patient safety initiatives.^1^ To address miscommunication and lack of teamwork skill, health care training programs implemented shared curricula encompassing diverse providers as members of a health care team. This shift in pedagogy was evidenced by the introduction of Interprofessional Education Collaborative (IPEC) Core Competencies for Interprofessional Collaborative Practice, which emphasize the attainment of safe, high-quality, patient-centered care through interprofessional learning.^2^ Interprofessional education (IPE) as defined by the IPEC (adapted from the WHO framework) occurs “when students from two or more professions learn about, from and with each other to enable effective collaboration and improve health outcomes.”^2^ This suggests the instructional delivery method of IPE to be interactive learning sessions. Thus, IPE is intended to prepare prepractice health care students for deliberate and effective collaborative practice when they enter the workforce.

One of the four core competencies of IPE is “Communicate with patients, families, communities, and professionals in health and other fields in a responsive and responsible manner that supports a team approach to the promotion and maintenance of health and the prevention and treatment of disease.”^2^ Interprofessional communication failures have been estimated to be a major factor in 60%-70% of serious health care–related accidents.^1–4^ During the transfer of information between providers, inadequate communication of vital information can occur.^2,5–8^ One intervention to improve safety during information transfer is a standardized communication protocol.^9–12^ For all health care providers, especially novices/learners, using a structured communication protocol (i.e., checklist) improves transfer of information and reduces the likelihood of omitting critical information expected by the recipient.^13–15^ An exemplar is the situational briefing tool Situation, Background, Assessment, Recommendation (SBAR).^11^ Its first noted use in health care was for rapid response teams at Kaiser Permanente in Colorado.^16^ SBAR has been adopted by many health care organizations and remains among the most popular handover mnemonic systems.^17,18^ The Institute for Health Improvement has suggested that SBAR be the model health care providers use to structure clinical communication.^19^ Additionally, SBAR is utilized as a communication tool in the TeamSTEPPS program developed in 2006 as a national standard for team training to improve quality, safety, and efficiency of health care.^20^ Pillars of TeamSTEPPS include leadership, situation monitoring, mutual support, and communication, which serve as the framework for the interprofessional training program described here.

We conducted a literature search of multiple databases, including PubMed, CINHAL, Embase, and Google Scholar, using the following keywords: *SBAR, communication,* and *assessment.* Two studies on using SBAR for prepractice students were discovered. One study reported higher SBAR communication skills after an educational program for senior-year nursing students.^21,22^ Its authors used a checklist of 12 items to measure the SBAR subscales and a 3-point scale for a global effectiveness rating (GER).^22^ Another study measured the posttraining effectiveness of SBAR among final-year medical students in a group simulation scenario that required making a referral phone call.^23^ These authors used a 20-point checklist for SBAR and a 5-point scale for the GER. Results showed higher scores for communication content and clarity after training.^23^

We built on these findings to design, implement, and evaluate our Interprofessional Team Training Day (ITTD) educational event. Previous work on multiple-week training sessions concentrated assessment on small sample sizes of one discipline using direct observation and nonvalidated checklists. Our approach used a previously validated checklist after an intensive SBAR training session. Use of one clinical vignette, recorded SBAR, and the validated scoring rubric provides a more objective assessment of student competency.

## Methods

To contribute to the field of prepractice IPE, we employed a valid and reliable measurement tool in a performance assessment context along with a controlled measurement of student learning gains to advance understanding of IPE instructional and assessment methods. ITTD was evaluated using a performance measurement by which students were rated on their use of SBAR during a simulated encounter—as opposed to measuring only knowledge on a written exam. Using the SBAR Brief Assessment Rubric for Learner Assessment (SBAR-LA; Appendix A),^24^ an instrument designed to measure SBAR skills, we aimed to rigorously establish whether ITTD was associated with improvement in students’ abilities at effective communication. We previously published in *MedEdPORTAL* on the design and testing of SBAR-LA and refer readers to that resource for validity support evidence, interrater reliability, and further information.^24^

The target learner audience was first-year health care professions students from the Emory University Schools of Medicine and Nursing enrolled in one of six educational programs: anesthesiologist assistant, genetic counseling, medicine, nursing, physical therapy, and physician assistant. Event organizers and small-group facilitators were faculty members from schools and departments of the Emory University Woodruff Health Sciences Center. Demographic and student performance data were collected as part of the educational event.

ITTD included a half-day of didactics, small-group discussion, and role-play scenarios using the SBAR tool. Learners were assessed on SBAR communication skills before and after the event (i.e., pre-/posttest). Student participation in the event and assessment was mandatory per all six program directors. The university's Institutional Review Board (IRB00091030) required electronic consent for students to opt in for or out of data analysis. Students completed the consent and attestation in the Canvas Learning Management System (Instructure). We have not included demographic or assessment data analysis in this publication for students who opted out.

### ITTD Educational Event

The original IPE curriculum was established at the institution in 2007. In 2008, it was officially titled ITTD and expanded to include all health professions students at the institution. ITTD curricular content was based on the IPEC Core Competencies^2^ and utilized TeamSTEPPS^20^ tools and strategies focused on the importance of clear, complete, concise communication in health care settings. A 20-minute plenary lecture provided a comprehensive overview of team concepts and communication skills (Appendix B). SBAR was highlighted as a key communication tool. Next, learners met in small interprofessional groups where they and faculty facilitators explored use of SBAR through case-based scenarios and role-play. Learners were asked to communicate critical information that required immediate attention and action concerning a patient's condition. Learners delivered or received patient information using SBAR and then received feedback from peers and facilitators. Two weeks before the event, faculty were provided a facilitator handbook (Appendix C).

### Learner Assessment

Learners were given an assignment in Canvas to perform a simulated SBAR exercise before and after training. Five days prior to the event, learners were emailed instructions, provided with a written clinical vignette, and directed to use SBAR to communicate critical health care information to another health care professional (Appendix D). Preevent assignment instructions were as follows: “Simply record your response to the best of your ability with respect to your prior knowledge and background experience” using the recording feature in Canvas that permitted students to submit an audio or video file. The same assignment was given to students at the conclusion of the event, and the second recording was due within 24 hours.

Raters (i.e., the authors) had previously been trained on use of the SBAR-LA rubric during the instrument testing phase.^24^ Recordings were distributed equally among raters, and two raters scored each recording. Raters scored the 10 subcategories on a 3-point scale (0 = *unsuccessful/did not attempt,* 1 = *attempted,* 2 = *successful*). For reliability purposes, ratings were dichotomized (0 = *did not attempt the skill,* 1 = *attempted and/or performed the skill*). Dichotomous subcategory scores were summed to give a maximum overall score of 10 points, with 1 point per subcategory: Situation (4 points), Background (3 points), Assessment (1 point), and Recommendation (2 points). Additionally, raters assigned a GER score on a 3-point scale: (0 = *not effective,* 1 = *moderately effective,* 2 = *very effective*). The analytic score for the 10 subcategories and the GER both represented raters’ assessment of the construct “communication” as defined by the SBAR framework. The dual-scoring method was utilized to confirm the target construct was being assessed, regardless of scoring technique.

### Statistical Analysis

A normalized gain score (*g*)^25^ was calculated for each learner using their SBAR-LA scores as [(postscore − prescore)/(10 − prescore)] where *g* was the percentage of skills that a learner had not demonstrated on the preevent measurement but subsequently demonstrated on the postevent measurement. Thus, the normalized gain score indicated how much of the material that a student did not know initially had been learned, presumably as the result of participating in the event.

Quantitative data analyses were conducted using SPSS v25 (IBM). We conducted three analyses to ascertain the value of ITTD. First, the normalized gain scores (*g*) were analyzed to determine the valence and magnitude of changes in score pre-/postevent.^25^ Positive, large values for *g* would suggest that learners had acquired new skills during ITTD that they had not exhibited previously. A sign test on the number of learners who increased their total scores, compared to those whose scores remained the same or decreased, was also conducted. Second, a repeated measures *t* test was used to compare GER pre-/postscores to ascertain whether the holistic performance scores of the learners had increased after ITTD. Third, a nonparametric sign test was used to scrutinize the 10 subcategories of SBAR-LA to identify skills on which learners demonstrated negligeable gains.^26^

## Results

A total of 651 learners attended ITTD, and 196 consented and participated in the assessment (Table 1). Learners (*n* = 196) who submitted pre- and postevent recordings in which both recordings were free from technical glitches (i.e., usable) and who consented to have their data used for program evaluation were included in the analyses (Table 2). Learners who attended and did not submit, submitted one recording, or submitted two but only one was usable were excluded. Despite the low assignment-completion rate, the response rates for the six programs were statistically equivalent.

**Table 1.**
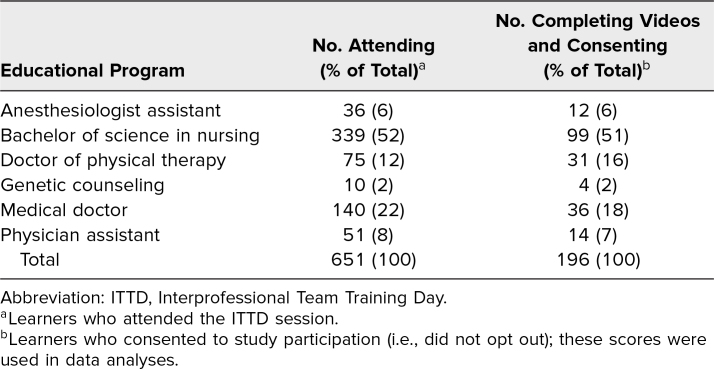
Composition of the ITTD Event and Research Study by Educational Program Affiliation

**Table 2.**
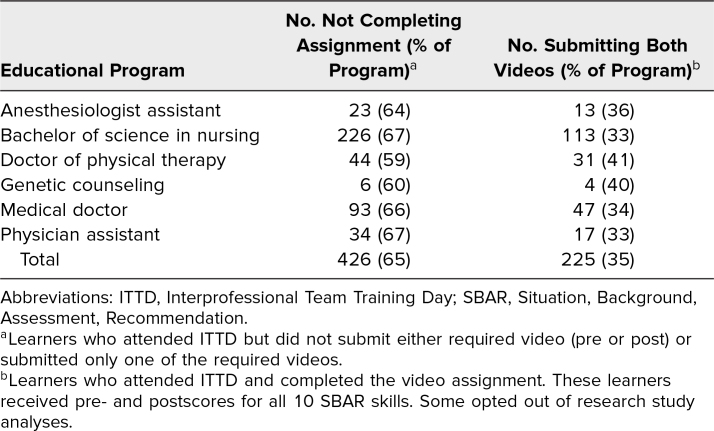
Rates of Assignment Completion for Each ITTD Educational Program

### Analysis One

Normalized gain scores, using each learner's summed score, were calculated and aggregated. Of the 196 learners, 118 (60%) increased their score; a sign test indicated a significant number of learners ( *p* = .001) had increased scores. This group that improved showed an average normalized gain of 58%. That is, of the skills not demonstrated initially, these learners were able to demonstrate more than half after the event. Of all learners, 20 (10%) had perfect postevent scores compared to three (2%) preevent. Pre-/postevent scores were identical for 58 learners (30%), while 20 (10%) had a lower postevent score. The average normalized gain score for all learners, including those whose score was 0 or negative, was 33%. Thus, any learner completing ITTD could be expected to have learned almost one-third of any skills that they were unable to perform prior to the event.

### Analysis Two

GERs were assigned on a scale of 0–2 points (0, 1, 2). Due to an error, 18 preevent GERs were not documented (*n* = 178). The GER mean was 1.2 (*SD* = 0.6) preevent and 1.5 (*SD* = 0.6) postevent. Using a repeated measures *t* test, we found the pre-/postevent GERs to be statistically different (*t* = −6.22, *p* < .001), with a Cohen's *d* effect size of 0.55. This corresponds to a moderate effect size, meaning the score increase is considered to have meaningful impact on actual practice.

Pre-/postevent GERs are displayed in a contingency table (Table 3), which illustrates that 107 learners (60%) had no change. Of this subgroup, five (5%) had ratings of 0 for both pre- and postevent measurements, 56 (52%) had ratings of 1 for both, and 46 (43%) had ratings of 2 for both. For the 71 (40%) of 178 learners who had changes pre-/postevent, 59 (83%) improved their rating by 1 or 2 points, and 12 (17%) earned a lower rating in the postevent. None of those whose rating decreased went down by more than 1 point.

**Table 3.**
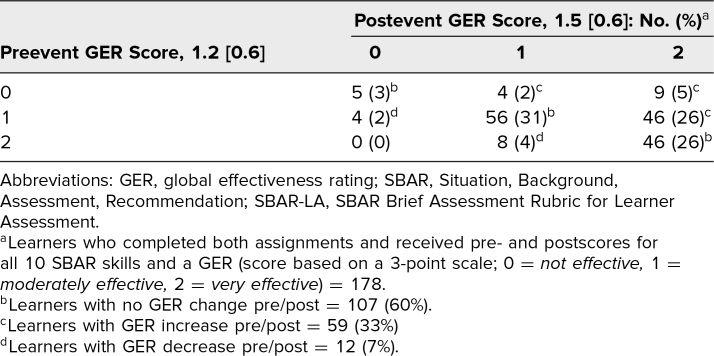
Contingency Table of Pre/Post GER Scores for SBAR Skills as Measured Using SBAR-LA

### Analysis Three

Because scores in SBAR-LA were made dichotomous, only learners who scored 0 on preevent skills were included in the calculation. For each of the 10 SBAR-LA skills, a sign test determined whether a statistically significant number of learners who had scored 0 on the preevent subsequently scored a 1 postevent. Learners whose score improved were considered positive value, and those whose score did not were negative value (Table 4). For five SBAR-LA skills (Provides patient name; States the context; States recent findings; Provides summary assessment of problem; Provides concrete suggested action), the sign test showed that the probability of frequency for positive values by randomness was below *p* = .05. Four skills (Identifies self; Provides a second patient identifier; Expresses situation, issue, concern; Provides facts only) had values that were not distinguishable from random possibility at *p* = .05. For one skill (Provides contact information), few learners demonstrated having acquired the skill postevent; the poor result was statistically likely to be attributable to an inherent flaw in the event itself.

**Table 4.**
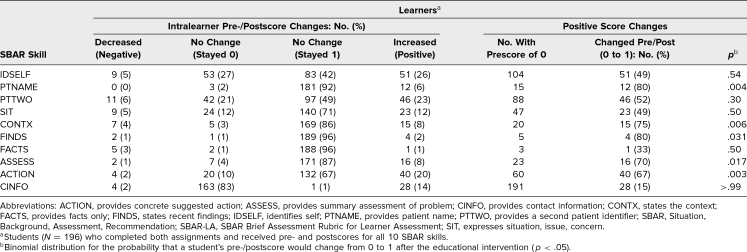
Frequency of Changes in Pre-/Postscores for SBAR Skills as Measured Using SBAR-LA

## Discussion

It is widely understood that communication breakdowns lead to medical errors. Structured tool use decreases the incidence of a health care provider-to-provider miscommunication. Our ITTD is grounded in widely accepted IPE models, specifically, IPEC Core Competencies and TeamSTEPPS.

Assessment is a continual challenge in medical education. We assessed students’ ability to effectively communicate with an innovative approach for assignment submission and scoring using the SBAR-LA rubric.^24^ The students received a clinical vignette and submitted an audio/video recording preevent. Using the same vignette, they submitted a second recording postevent. Although the scenario involved a nurse calling a doctor, students were told the circumstances could be applicable to other sender/receiver pairs (student to physician, physical therapist to nurse, nurse to nurse, etc.). Designing scenarios for each potential learner dyad may be feasible; however, our learning objective was to demonstrate effective use of SBAR. The one-scenario design minimized variability in scoring and comparing student performance. It will also improve reproducibility for institutions that adopt this resource.

Two raters independently scored the recordings. Maximum clip time was 2 minutes. Canvas allowed playback at 0.5, 1.0, 1.5, and 2.0 times the recording speed, so clear audio clips could be sped up, and noisy clips could be replayed or slowed down. Average score time per clip was not tracked, although we estimated the total time per rater to score 10 clips was 15 minutes, equivalent to 40 clips per hour. Scoring time was minimized by having only one reviewer score the single scenario.

Analysis of skills measured by SBAR-LA revealed a statistically significant improvement for five of 10 skills (Table 4). Considering the aim of a structured communication tool, these skills could be equated with a successful educational program. Four skills showed no change that could be differentiated from chance. We believe that these skills, although important, were perceived as lower value compared to relating facts and action plan. Furthermore, we noted there was no change in the skill Provides contact information. We believe that this may have been due to a lack of addressing this item in the curriculum or because the vignette did not provide a callback number to use and that students may have been reluctant to use their own number or invent one.

Our results revealed that 60% of learners demonstrated improvement. The evidence supports the idea that the event enhanced students’ skills at communicating patient information in a simulated scenario—a step beyond simply measuring their knowledge about communication. This aligns with Kirkpatrick's level 2: “The degree to which participants acquire the intended knowledge, skills, attitude, confidence, and commitment based on their participation in the training.”^27^ Although behavior in the simulated environment is more authentic than a written exam, our future plan is to measure students’ workplace performance at Kirkpatrick level 3: “The degree to which participants apply what they learned during training when they are back on the job.”^27^ The students in our training demonstrated pre/post behavioral changes that should be readily transferable to clinical practice, and further assessment of the trainees’ communication in clinical settings should be used to advance the evaluation of our training approach.

### Study Limitations

This study was conducted with one interprofessional cohort of learners at a single institution. Replication in a broader variety of contexts is necessary to further substantiate our findings.

Embedded in IPE events is the fact that each discipline has its own distinct educational needs. The ITTD planning committee coordinates around schedules of the six participating programs. There is invariably some resistance from faculty and students about why they need to spend an afternoon of learning time for IPE. One study examined combined courses for IPE and discussed institutional barriers that mirror our experience. Its authors encountered “obstacles of combining curricula from different faculties, organizational aspects e.g., lack of infrastructures to accommodate all students, difficulty in coordinating rotations, time constraints, monetary constraints and deanery or political barriers.”^28^

A disconcerting number of students did not submit the assigned recordings (Table 2). The overall completion rate was 35%. Nursing (33%) and physician assistant (33%) had the lowest completion rates, while physical therapy (41%) and genetic counseling (40%) had the highest. For some of our programs, ITTD provided students with required graduation credits. For others, although ITTD was required, there were no mechanisms to enforce participation. While the assignment was also required, there were no sanctions for incompleteness. Analysis was also hampered by many students who did not consent to our use of their scores. We suspect that students misunderstood how their recordings would be used or who would have access to view their recordings. In subsequent years, we will affect submission rate through transparency and reminders to students and grade/score and professionalism reports to program directors. Perhaps as clinical regard for IPE becomes more commonly recognized and practiced by health care professionals, there may be a top-down push for greater academic accountability.

Recordings were scored in a random order by raters such that pre-/postevent clips were not scored sequentially. We could not remove/anonymize the assignment title (i.e., pre-/posttest). Thus, while raters were asked to ignore the title, this may have produced bias in scoring decisions.

Finally, we did not design our curriculum to assess how the learners perceive their own performances as compared to the assessment by trained raters. However, learners did have access to view their recordings in Canvas. We think self-assessment by learners (subjective or objective) could provide informative data on how they feel about their gain in skills, the design of the event, and the realism of the vignette. This is one focus of our future scholarly work.

### Conclusion

While the ability of health care providers to work seamlessly together leads to better outcomes, determining whether health care learners are being effectively taught the skills they need remains difficult. Although health professions programs and educators can teach SBAR skills, there are limited methods to assess learning or impact. Using the SBAR-LA rubric to measure student performance before and after an IPE event, we found that SBAR skills improved following ITTD—a short-duration, IPE, SBAR-based, educational event.

## Appendices


SBAR-LA Rubric.docxITTD Lecture.pptxITTD Faculty Facilitator Handbook.pdfLearner SBAR Assignment.docx
*All appendices are peer reviewed as integral parts of the Original Publication.*
