# Supplementary material for: Situation, Background, Assessment, Recommendation (SBAR) Education for Health Care Students: Assessment of a Training Program
Source: MedEdPORTAL. 2023 Jan 3;19:11293. doi: 10.15766/mep_2374-8265.11293 (PMC9807695; doi:10.15766/mep_2374-8265.11293)
Supplement: Supplementary file 1 — SBAR-LA Rubric.docxITTD Lecture.pptxITTD Faculty Facilitator Handbook.pdfLearner SBAR Assignment.docx [file mep_2374-8265.11293-s001.zip › B. ITTD Lecture.pptx]

## Slide 1
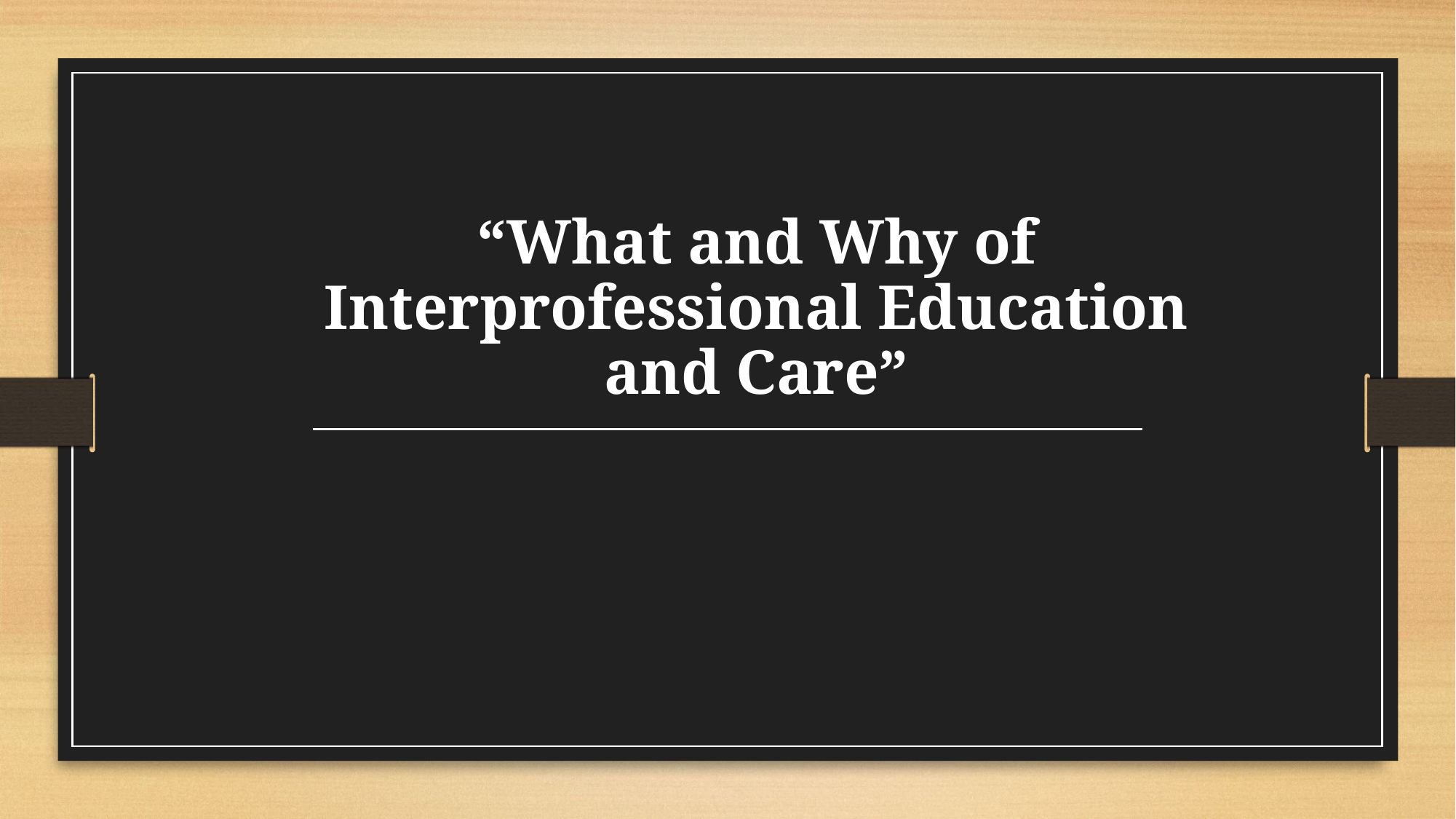

# “What and Why of Interprofessional Education and Care”

## Slide 2
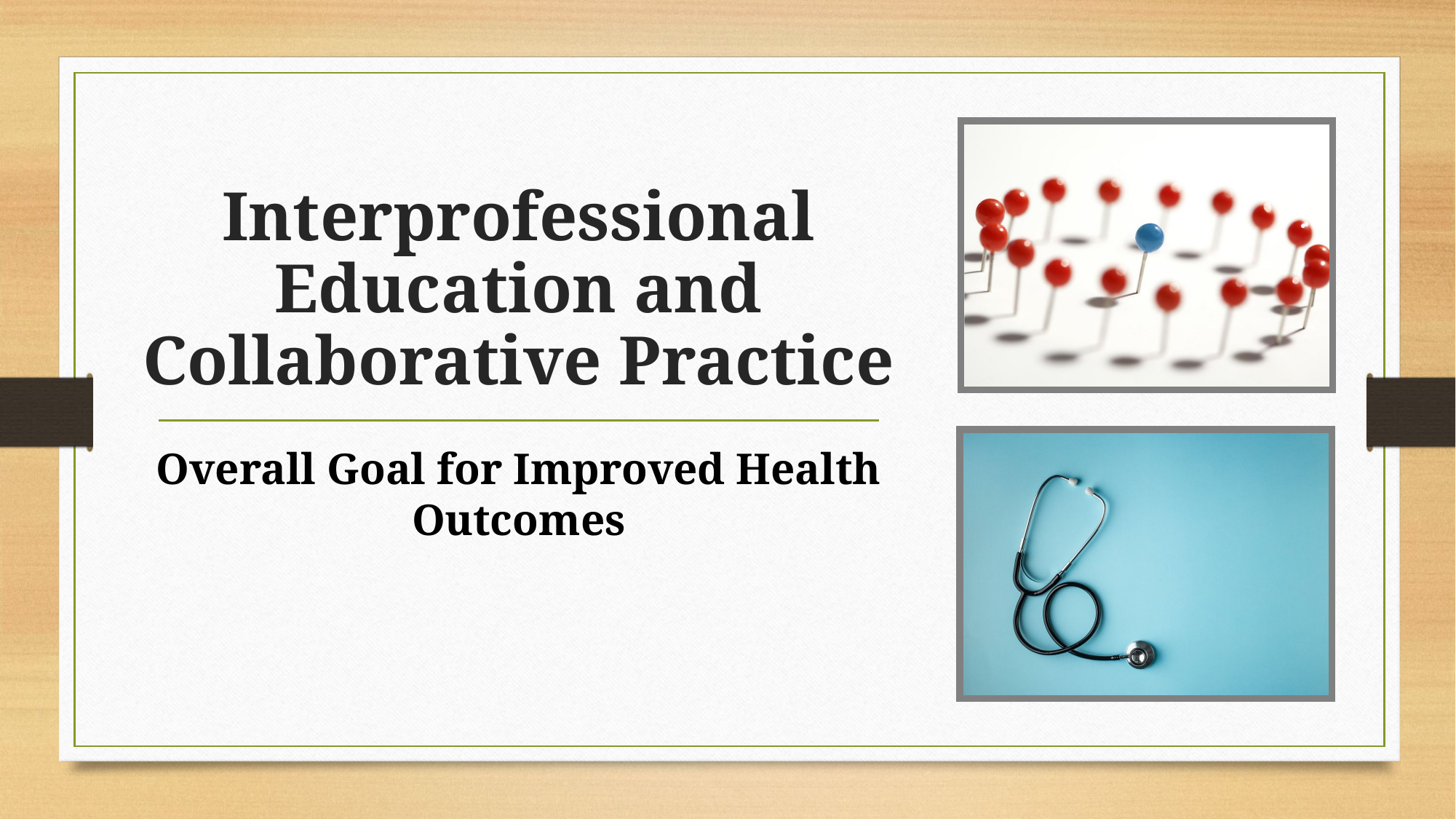

# Interprofessional Education and Collaborative Practice
Overall Goal for Improved Health Outcomes

## Slide 3
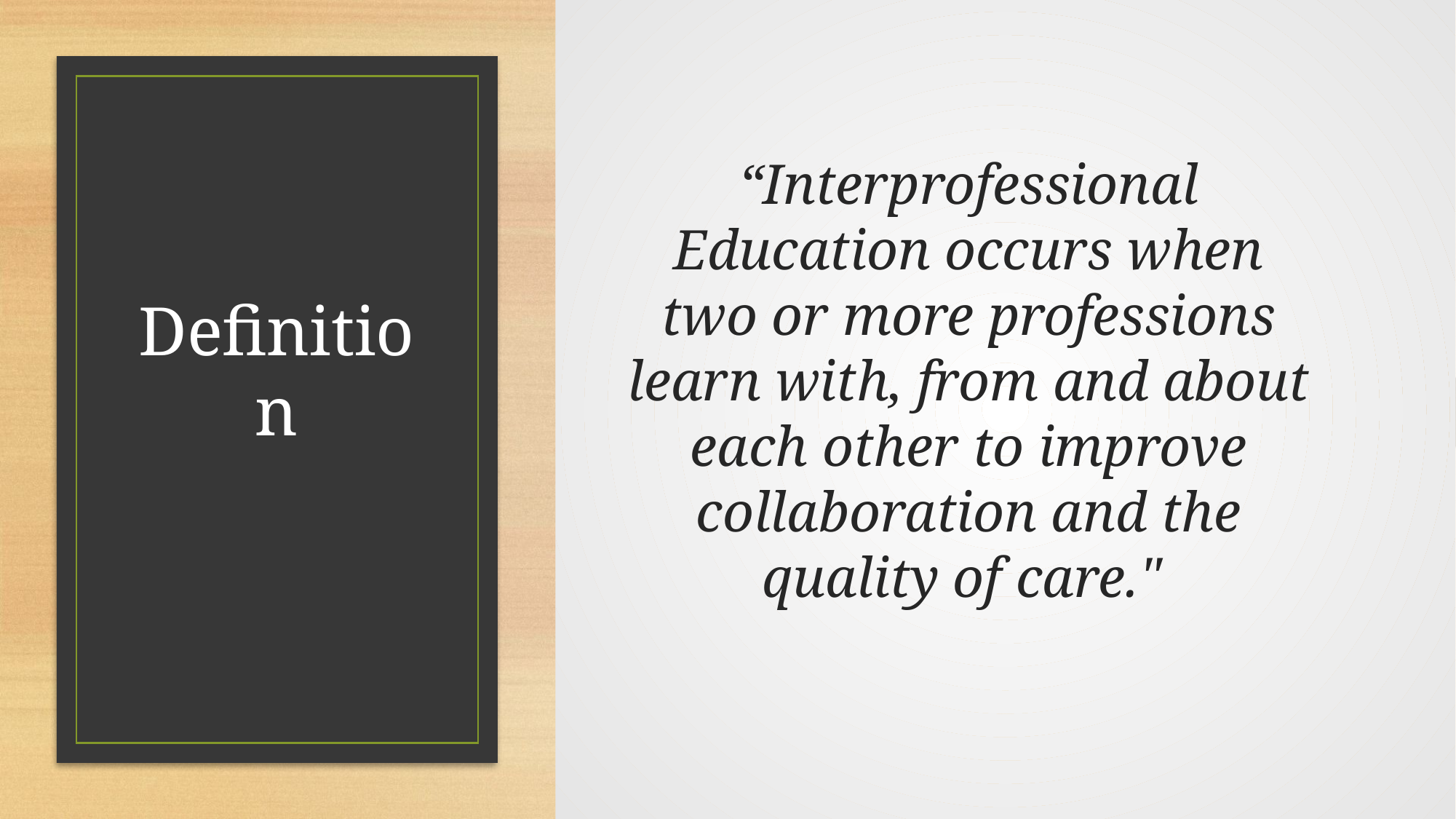

“Interprofessional Education occurs when two or more professions learn with, from and about each other to improve collaboration and the quality of care."
# Definition

## Slide 4
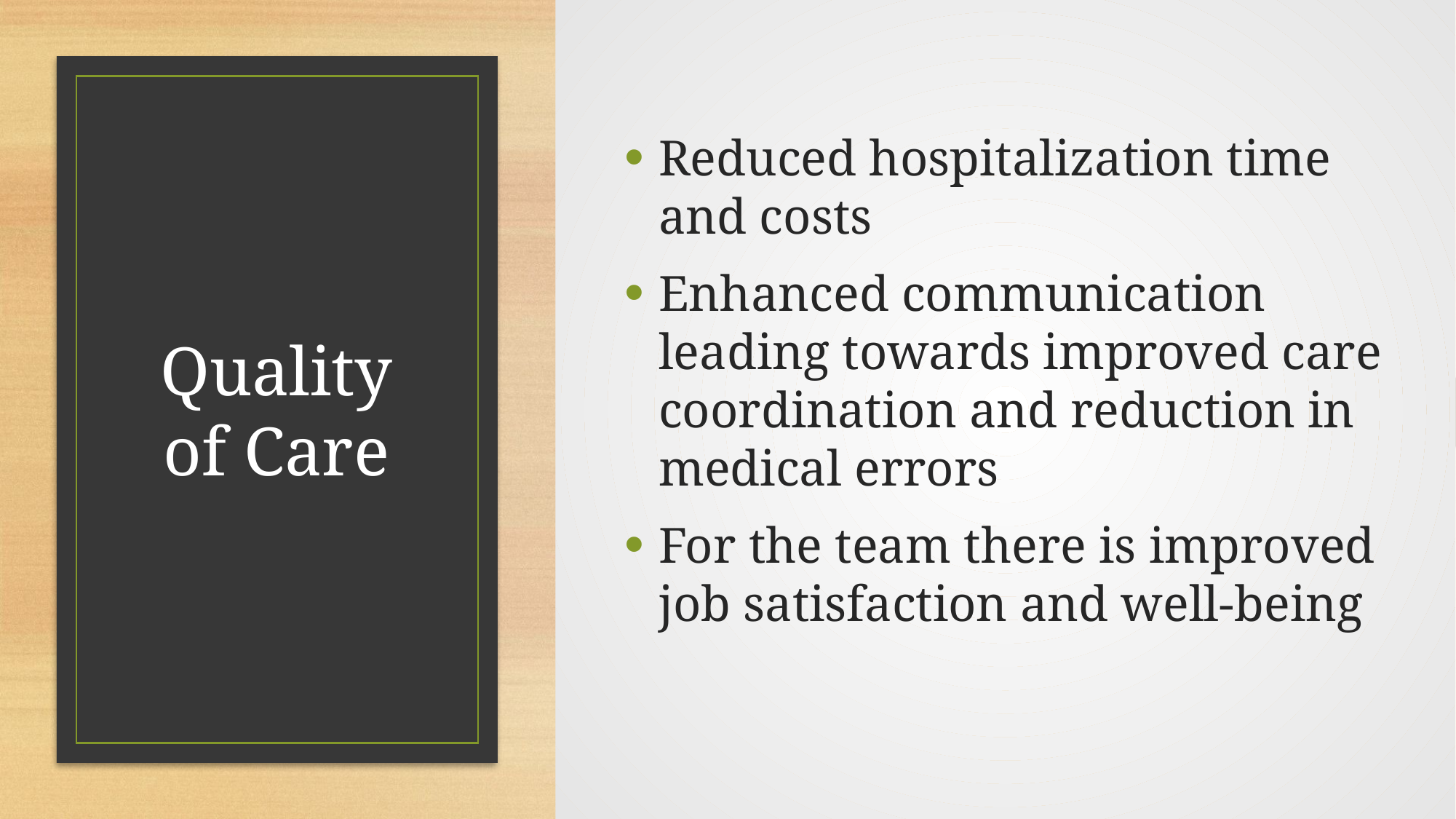

Reduced hospitalization time and costs
Enhanced communication leading towards improved care coordination and reduction in medical errors
For the team there is improved job satisfaction and well-being
# Quality of Care

## Slide 5
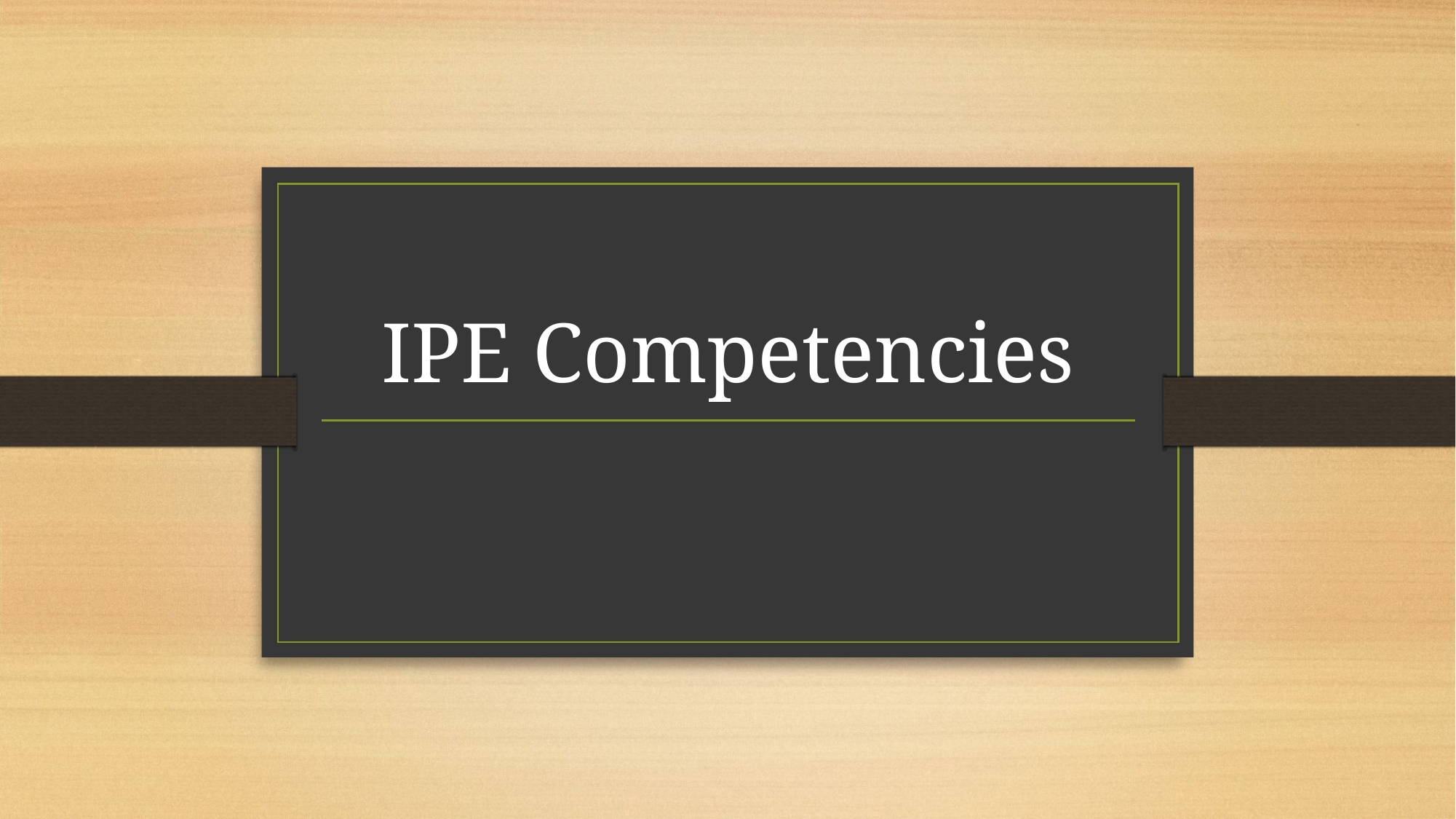

IPE Competencies

## Slide 6
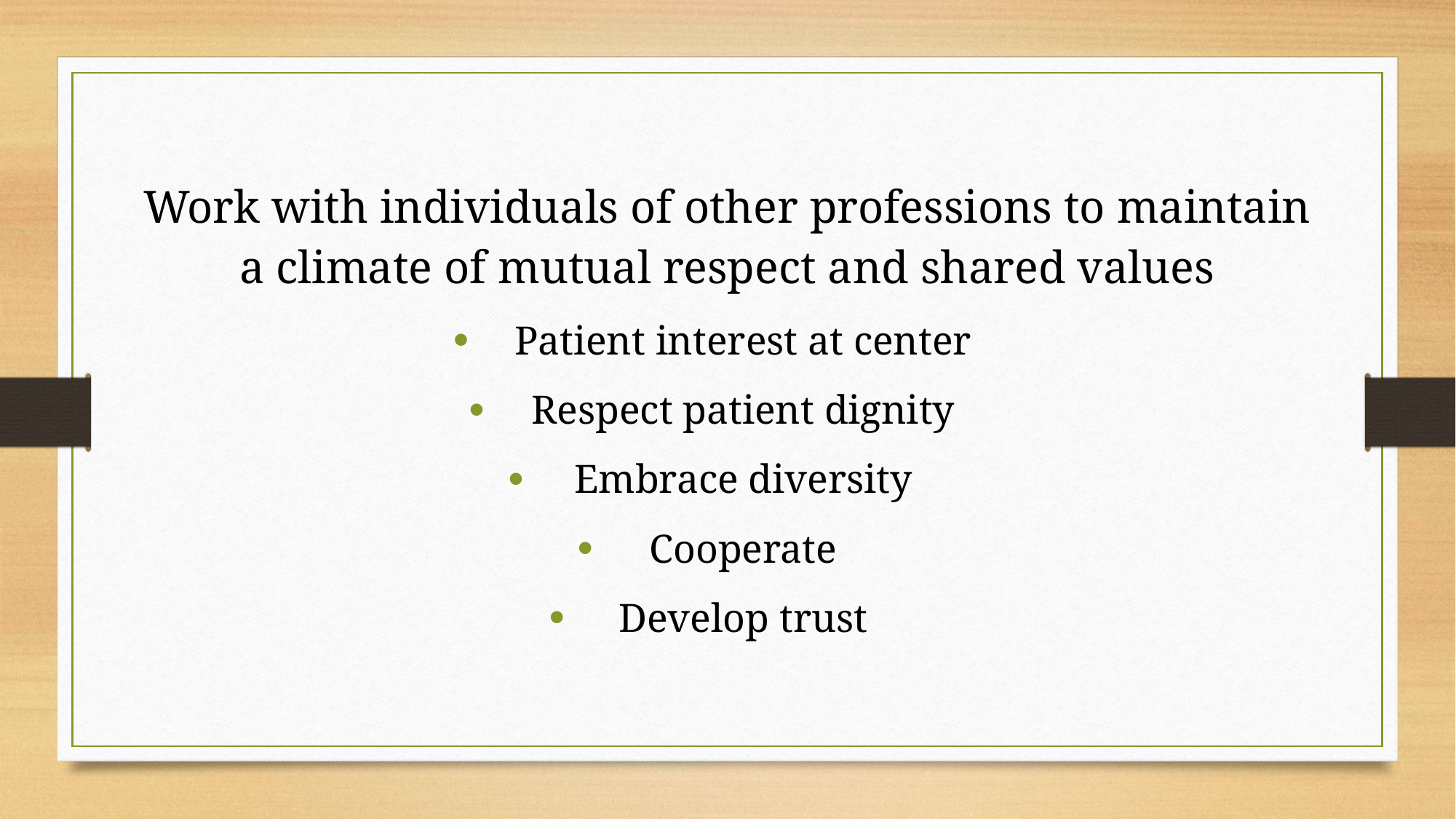

Work with individuals of other professions to maintain a climate of mutual respect and shared values
Patient interest at center
Respect patient dignity
Embrace diversity
Cooperate
Develop trust

## Slide 7
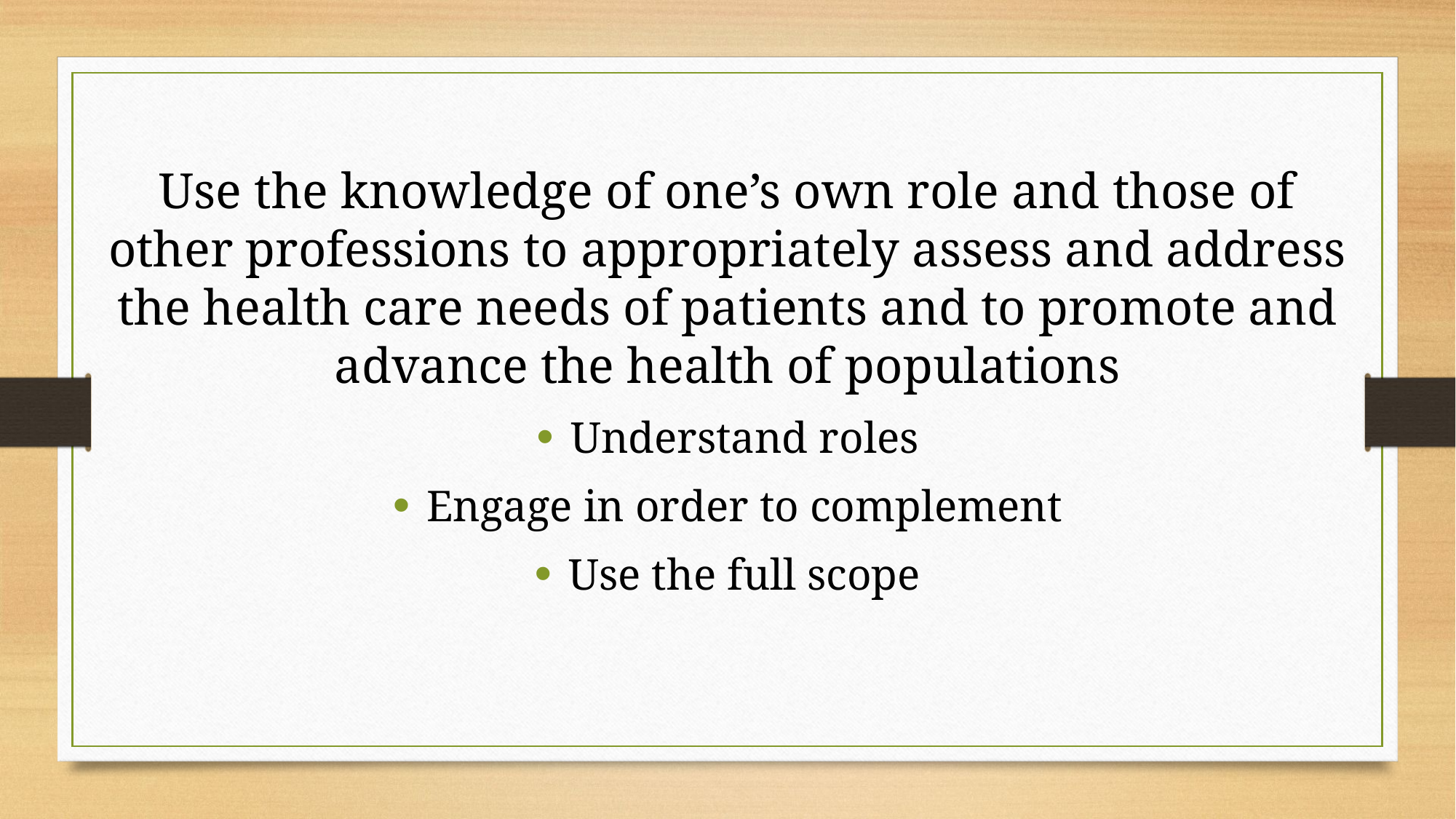

Use the knowledge of one’s own role and those of other professions to appropriately assess and address the health care needs of patients and to promote and advance the health of populations
Understand roles
Engage in order to complement
Use the full scope

## Slide 8
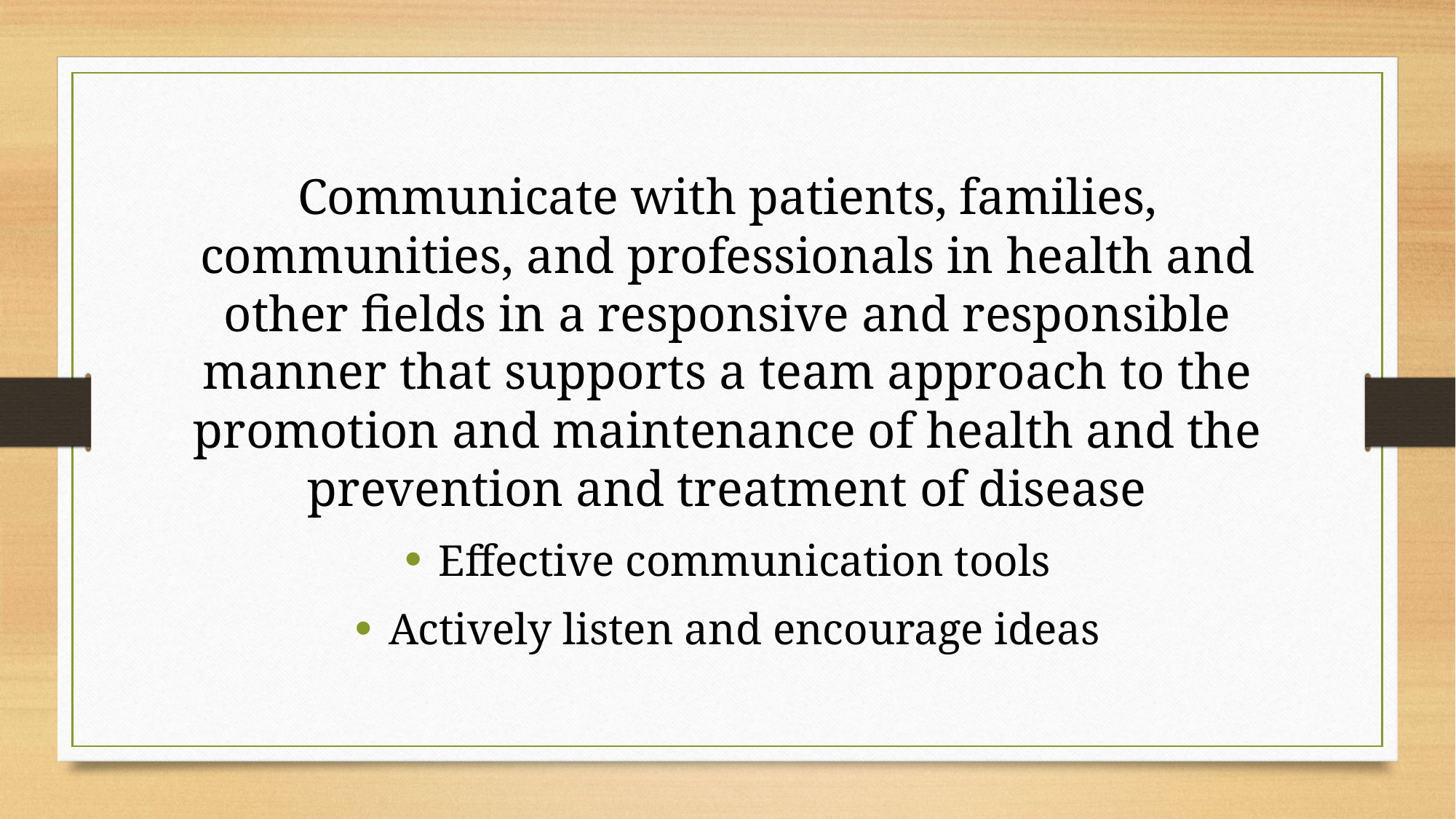

Communicate with patients, families, communities, and professionals in health and other fields in a responsive and responsible manner that supports a team approach to the promotion and maintenance of health and the prevention and treatment of disease
Effective communication tools
Actively listen and encourage ideas

## Slide 9
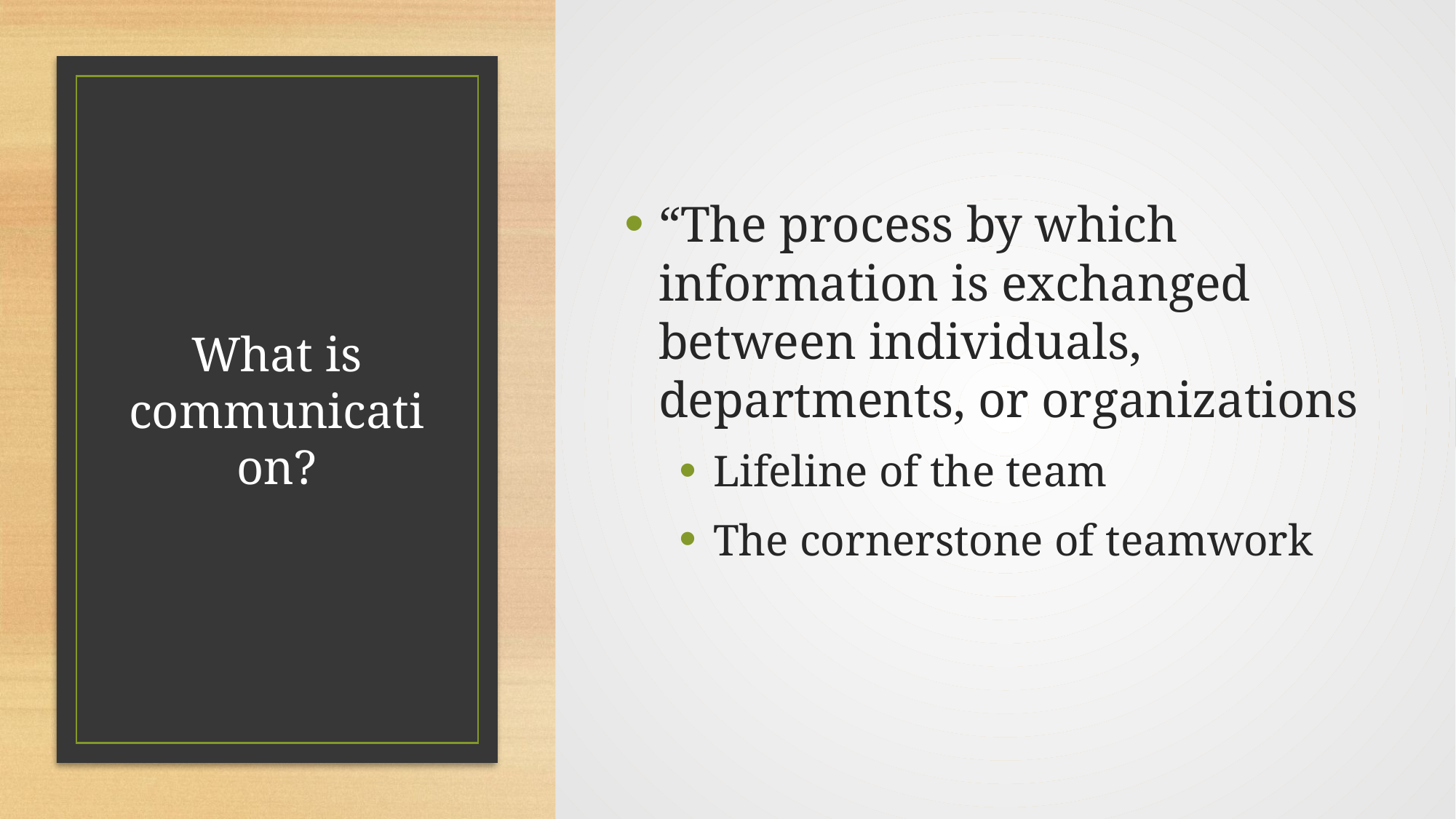

“The process by which information is exchanged between individuals, departments, or organizations
Lifeline of the team
The cornerstone of teamwork
What is communication?

## Slide 10
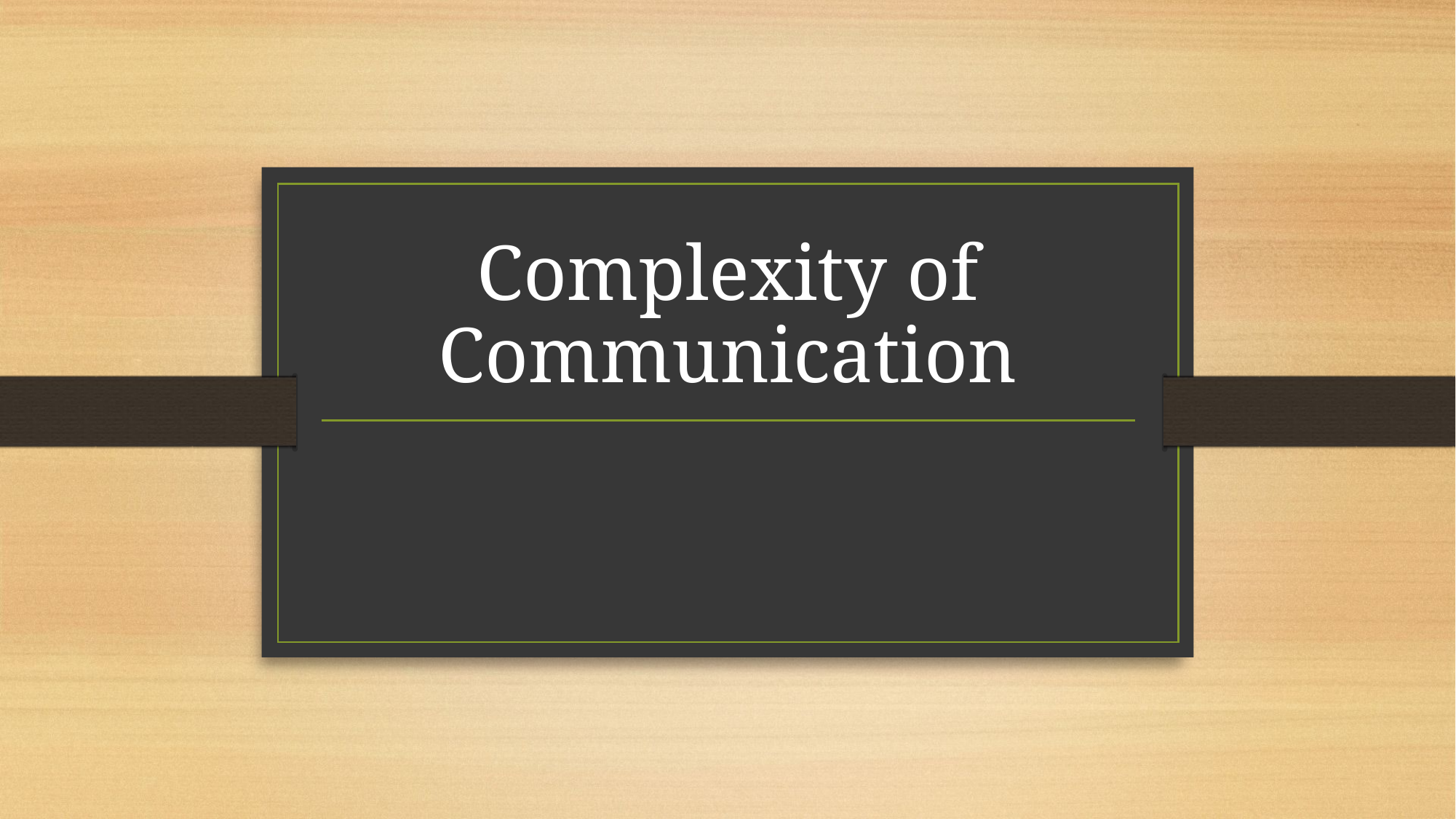

Complexity of Communication

## Slide 11
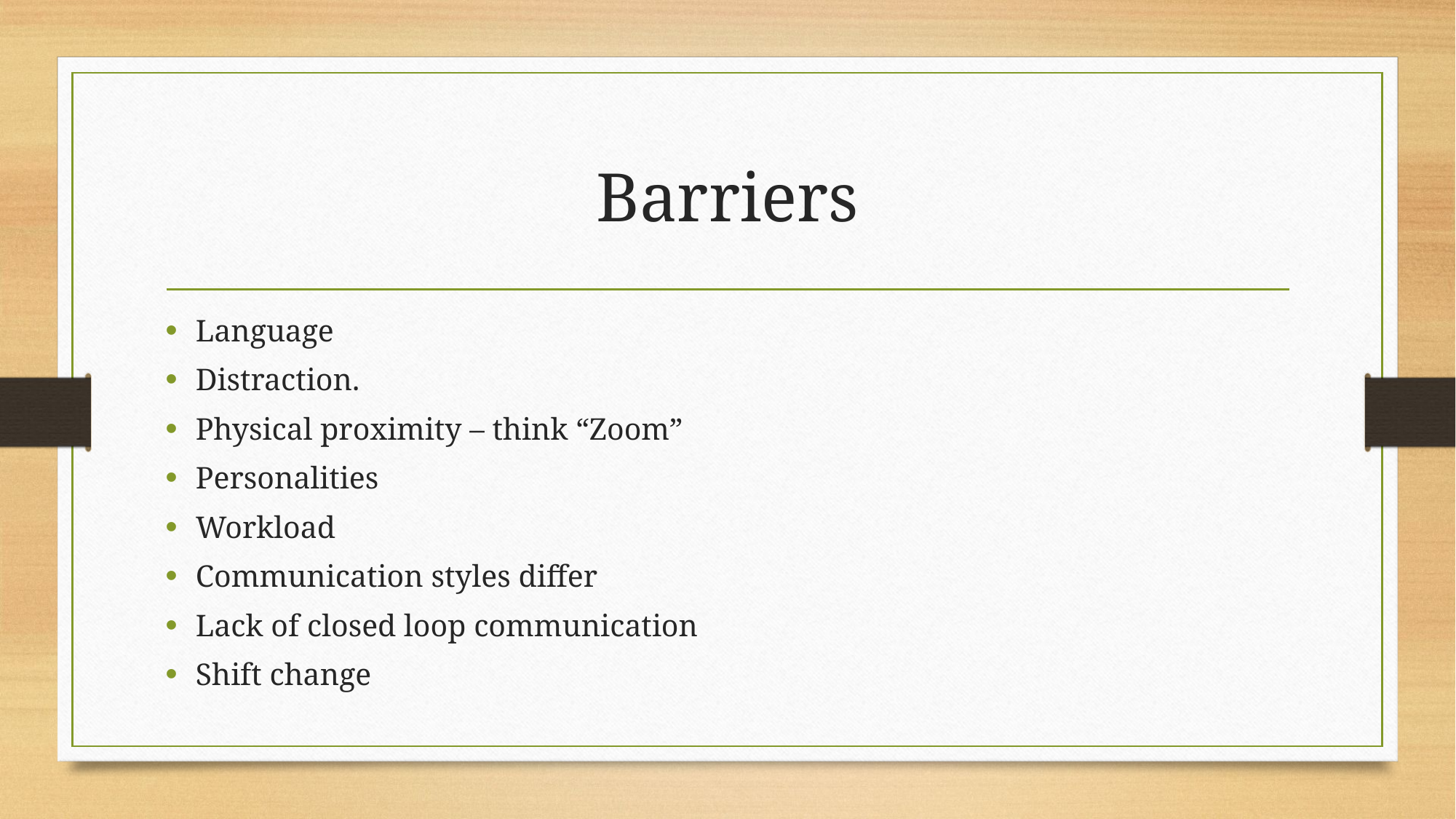

# Barriers
Language
Distraction.
Physical proximity – think “Zoom”
Personalities
Workload
Communication styles differ
Lack of closed loop communication
Shift change

## Slide 12
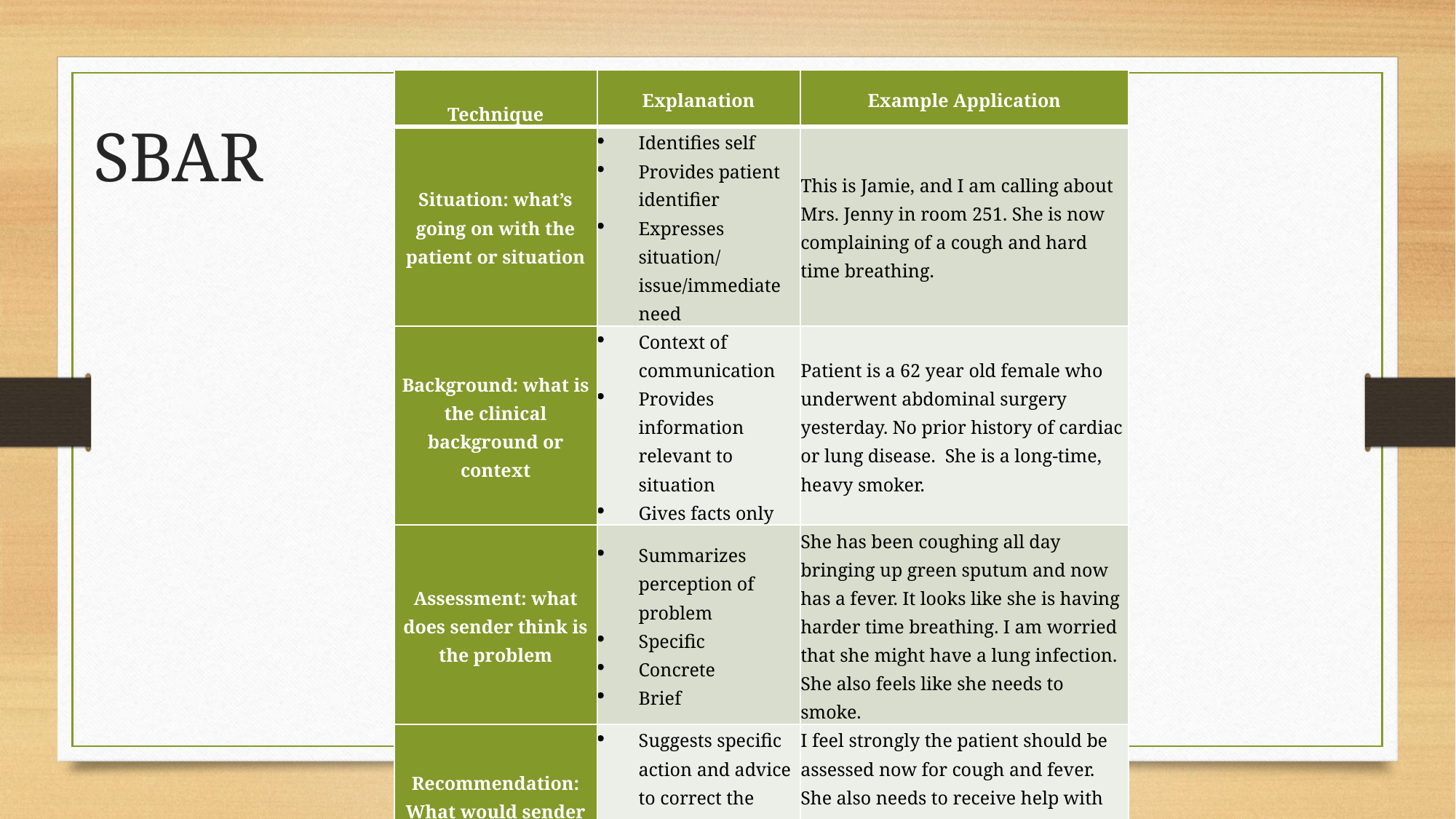

| Technique | Explanation | Example Application |
| --- | --- | --- |
| Situation: what’s going on with the patient or situation | Identifies self Provides patient identifier Expresses situation/ issue/immediate need | This is Jamie, and I am calling about Mrs. Jenny in room 251. She is now complaining of a cough and hard time breathing. |
| Background: what is the clinical background or context | Context of communication Provides information relevant to situation Gives facts only | Patient is a 62 year old female who underwent abdominal surgery yesterday. No prior history of cardiac or lung disease.  She is a long-time, heavy smoker. |
| Assessment: what does sender think is the problem | Summarizes perception of problem  Specific Concrete Brief | She has been coughing all day bringing up green sputum and now has a fever. It looks like she is having harder time breathing. I am worried that she might have a lung infection. She also feels like she needs to smoke. |
| Recommendation: What would sender do to correct the problem | Suggests specific action and advice to correct the problem Provides contact information for follow up | I feel strongly the patient should be assessed now for cough and fever. She also needs to receive help with smoking cessation. Are you available to come in? Can you help her access the national quit line? I can be reached at 404-867-5309. |
SBAR

## Slide 13
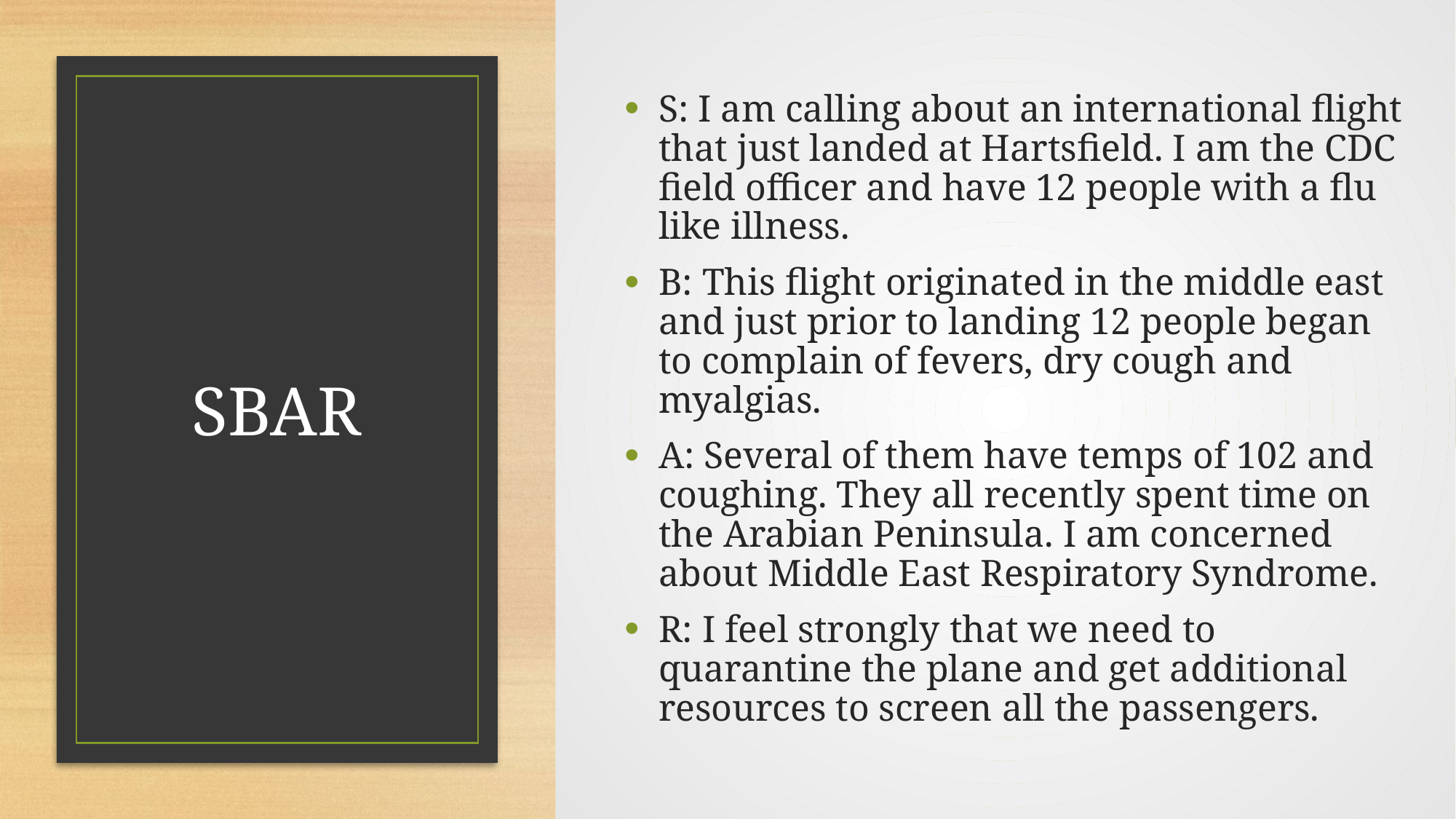

S: I am calling about an international flight that just landed at Hartsfield. I am the CDC field officer and have 12 people with a flu like illness.
B: This flight originated in the middle east and just prior to landing 12 people began to complain of fevers, dry cough and myalgias.
A: Several of them have temps of 102 and coughing. They all recently spent time on the Arabian Peninsula. I am concerned about Middle East Respiratory Syndrome.
R: I feel strongly that we need to quarantine the plane and get additional resources to screen all the passengers.
SBAR

## Slide 14
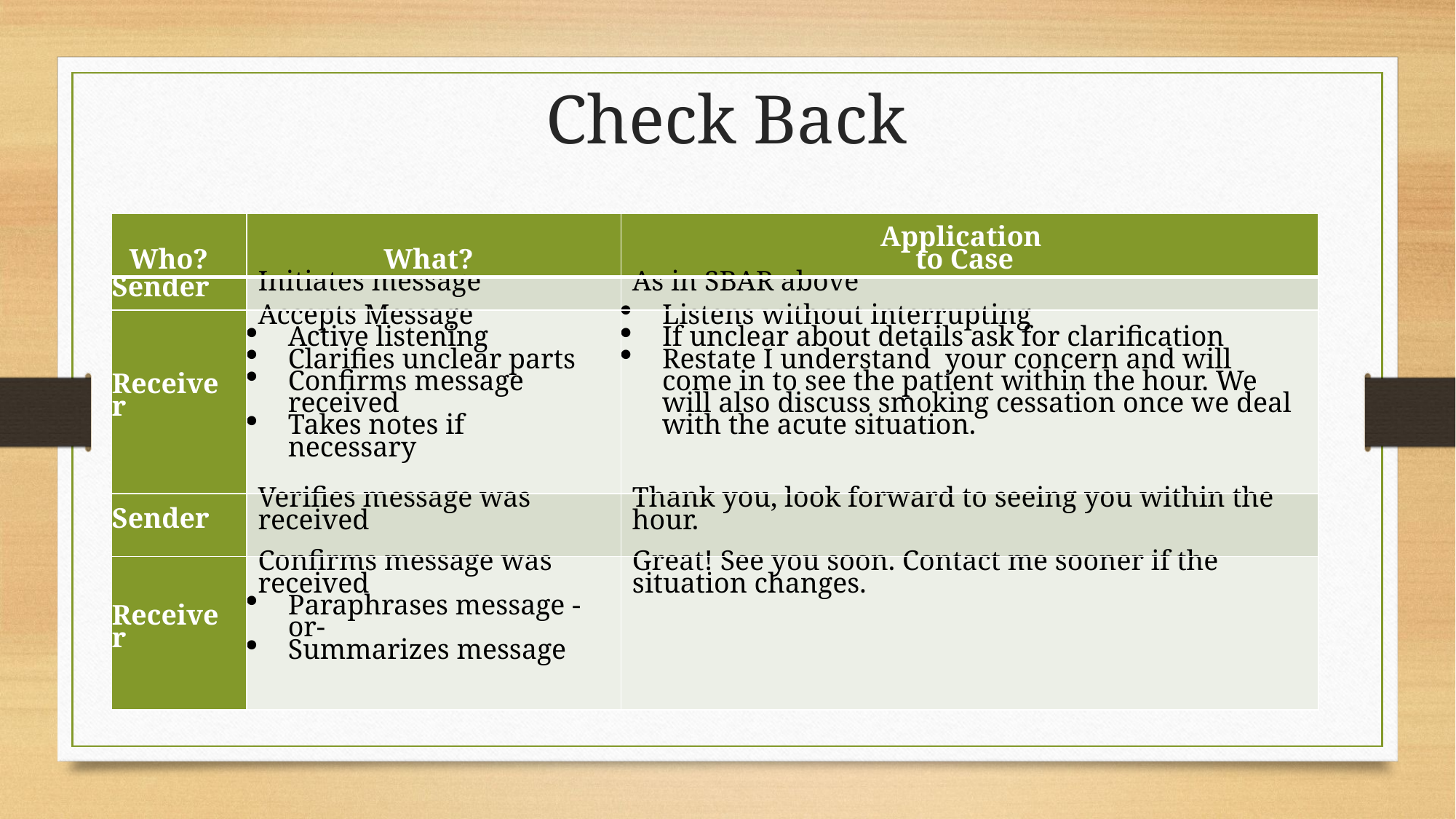

# Check Back
| Who? | What? | Application to Case |
| --- | --- | --- |
| Sender | Initiates message | As in SBAR above |
| Receiver | Accepts Message Active listening Clarifies unclear parts Confirms message received Takes notes if necessary | Listens without interrupting If unclear about details ask for clarification Restate I understand your concern and will come in to see the patient within the hour. We will also discuss smoking cessation once we deal with the acute situation. |
| Sender | Verifies message was received | Thank you, look forward to seeing you within the hour. |
| Receiver | Confirms message was received Paraphrases message -or- Summarizes message | Great! See you soon. Contact me sooner if the situation changes. |

## Slide 15
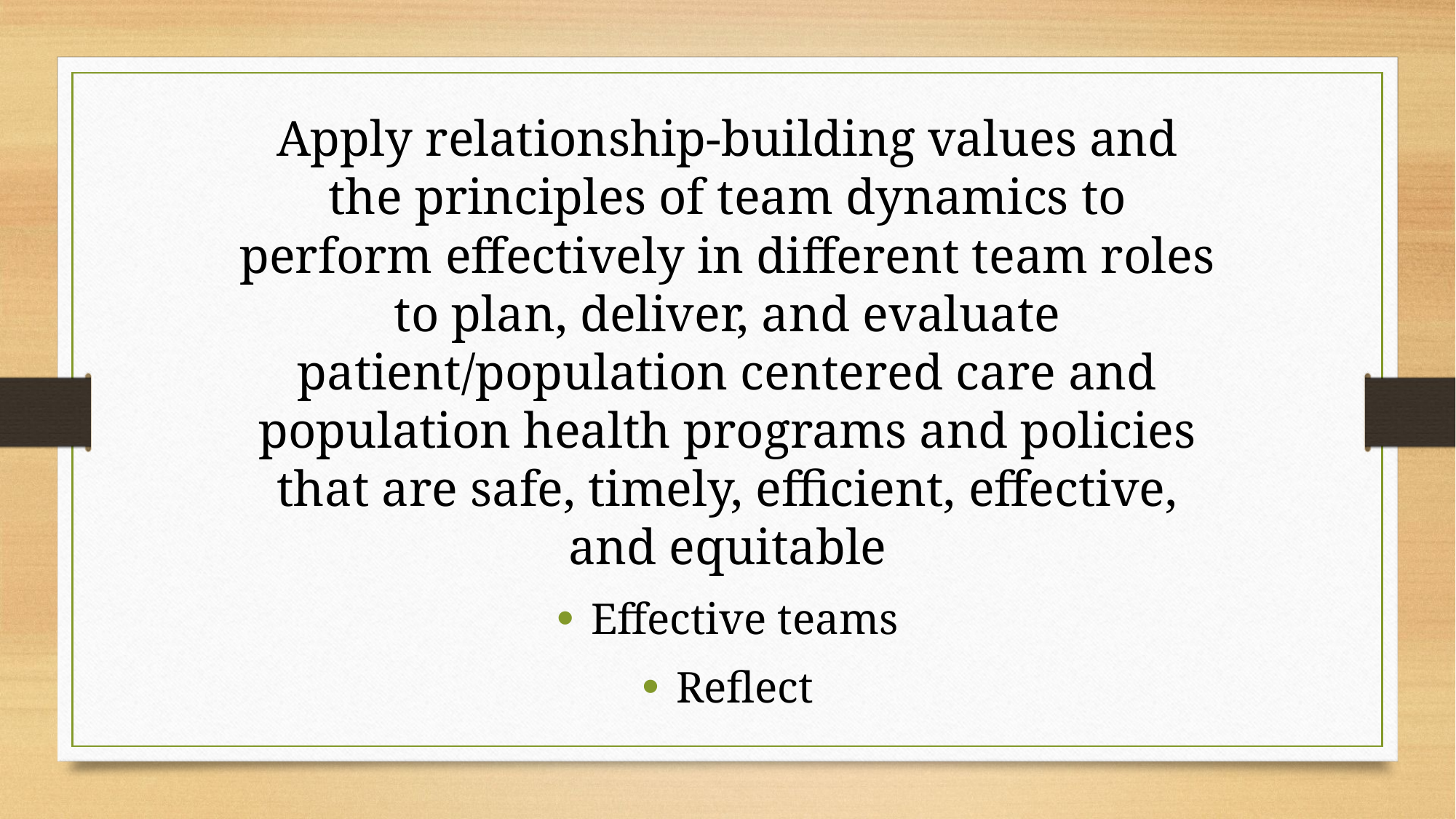

Apply relationship-building values and the principles of team dynamics to perform effectively in different team roles to plan, deliver, and evaluate patient/population centered care and population health programs and policies that are safe, timely, efficient, effective, and equitable
Effective teams
Reflect

## Slide 16
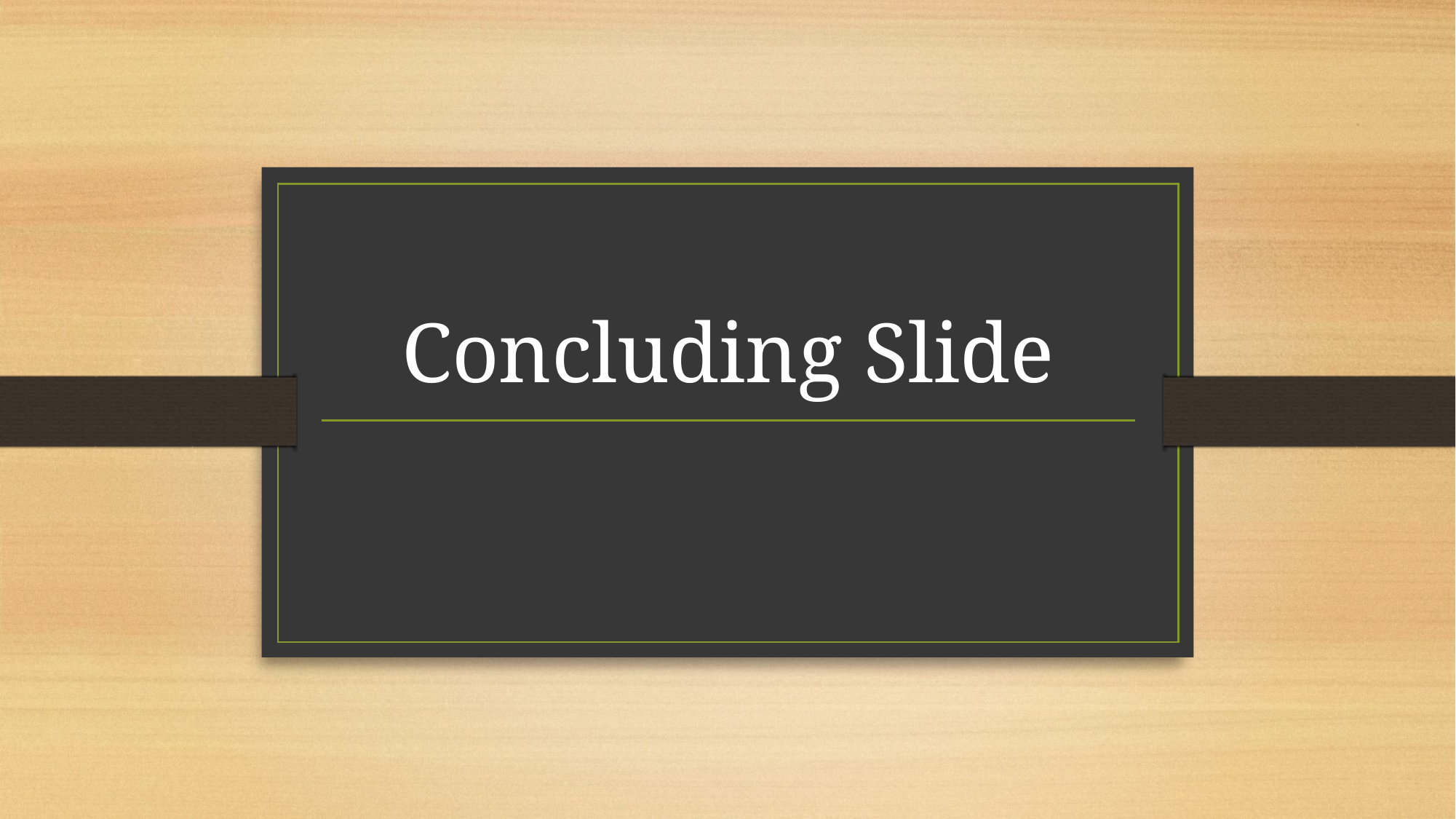

# Concluding Slide
