# Supplementary material for: Situation, Background, Assessment, Recommendation (SBAR) Education for Health Care Students: Assessment of a Training Program
Source: MedEdPORTAL. 2023 Jan 3;19:11293. doi: 10.15766/mep_2374-8265.11293 (PMC9807695; doi:10.15766/mep_2374-8265.11293)
Supplement: Supplementary file 1 — SBAR-LA Rubric.docxITTD Lecture.pptxITTD Faculty Facilitator Handbook.pdfLearner SBAR Assignment.docx [file mep_2374-8265.11293-s001.zip › C. ITTD Faculty Facilitator Handbook.pdf]

Interprofessional Team Training Day

Facilitator Handbook

DATE

Learning Objectives

First Year Health Science Students will:

- Describe the complementary roles played by diverse health professionals
- Explain how inter-professional teams improve the health of individuals and communities
- Demonstrate effective inter-professional communication skills

Overview of Learning Day

Facilitator Schedule

(Just in Time Facilitator Training)

At our institution we incorporated facilitator training during the Introductory Didactic Lecture for the students. In previous years, we had separate facilitator training sessions (3-4) at various times in the weeks preceding the team training program.

Student Schedule

We have left the times to demonstrate how we split the large cohort into two for the lecture and staggered the times. This can be adjusted for the specific needs of your program.

**Group A** timeline	**Activity** (time allotted)	**Group B** timeline
7:45	Arrival and Check-in (15 min)	9:15
8:00-8:30	Introductory Didactic Lecture (30 min)	9:30-10:00
8:30-8:45	Transfer to Small Groups (15 min)	10:00-10:15
8:45-10:10	Small Groups (1hr 20 min)	10:15-11:40

Small Group Activities

Recommend time allocations

Step	Time (min)	Activity
1	5	Student sign in and facilitator introduction
2	25	Ice breaker/introductions and role discussions
3	5	Review communication guides and example
4	25	Case activity (4 Groups, 4 Cases)
5	25	Case presentations and discussion (all together)

Ice Breakers: Small Group

Facilitator Introduction

(1-2 Minutes)

- Briefly summarize your profession and role as it relates to patient care
- Share a memorable "teamwork" or "communication" experience

Icebreaker: Interprofessional Exchange

(5-8 Minutes)

Have students break up into small groups of 2-3 people *from different disciplines* and introduce themselves to each other.

Discuss in small groups: (Facilitator gives questions verbally)

- How each chose their profession and what is unique about their profession (vs. others)
- What classes they are most looking forward to and why
- How they understand the relationships between their profession and others

Individual Introductions

(12-15 minutes)

Based on the discussion above, each student will:

- Introduce one small group peer to the larger group (1 minute/student)
- Identify any "surprises" about the other student's profession

Communication Guides for Students

Instructions: Pass out guides and paper copies to write on for case activity. Briefly review with students SBAR/Check-back components. Discuss example on next page. (3-5 min)

SBAR Guide for Case Discussion (Sender)

Technique	Explanation	Application to Case
**S**ituation	• 8-12 seconds<br>• Grabs listener's attention<br>• Convey immediate need	
**B**ackground	• Context of communication<br>• What is relevant to situation<br>• Give facts only	
**A**ssessment	• Summarize perception of problem<br>• Specific<br>• Concrete<br>• Brief	
**R**ecommendation	• Specific action you suggest<br>• Advice to correct the problem	

Remember:

1. SBAR is a 20-60 second very brief communication tool
2. It summarizes the *most relevant* information, *not all* information

Check-Back Guide for Cases (Sender & Receiver)

Who?	What?	Application to Case
Sender	*Initiates* message	*As in SBAR above*
Receiver	*Accepts* Message<br>• Active listening<br>• Clarifies unclear parts<br>• Confirmations message received<br>• Takes notes if necessary	
Sender	*Verifies* message was received	
Receiver	*Confirms* message was received<br>• *Paraphrases* message -or-<br>• *Summarizes* message	

Example of SBAR Guide

**Situation-What is going on with the patient?**<br>*"I am calling about Mrs. Joseph in room 251. Chief complaint is shortness of breath of new onset."*
**Background-What is the clinical background or context?**<br>*"Patient is a 62 year old female post-op day one from abdominal surgery. No prior history of cardiac or lung disease. She is a long-time, heavy smoker."*
**Assessment-What do I think the problem is?**<br>*"Breath sounds are decreased on the right side with acknowledgement of pain. Would like to rule-out pneumothorax and help her to stop smoking."*
**Recommendation-What would I do to correct it?**<br>*"I feel strongly the patient should be assessed now and receive help with smoking cessation. Are you available to come in? Can you help her access the national quit line?"*
https://www.ahrq.gov/teamstepps/instructor/essentials/pocketguide.html#sbar

Case Activity (Small Groups)

(Student –Led: Total 25 min)

Instructions:

- Divide students into 4 groups making sure the professions are intermixed.
- Each small group receives one of the 4 different case scenarios (pages 7-14).
- Have one person in each of the 4 groups read the case aloud and allow students to ask peers clarification questions if unfamiliar with the medical issues.
- Using the SBAR guide to communication, students discuss and fill in the blanks for S, B, A, R based on their case. Similarly for the Check-Back guide.
- Have students consider/briefly discuss the questions that follow the case under the four categories of: mutual respect, roles, communication, and teams.

Case Presentations and Discussion (Whole Group)

(Facilitator-Led: Total 25 min)

Instructions:

- Once the 4 groups have completed the SBAR & Check-Back Guides and review of the questions, bring them back together as one large group.
- Ask the 3 volunteers from each group to present their case:
  - One reads the case
  - One presents the SBAR solutions
  - One presents the Check-Back solutions

- Discuss the following points about the SBAR/Check-Back communication as a large group:
  - *What was your understanding of what was communicated?*
  - *Which points of the communication do you feel were most effective?*
  - *How did the sender ensure the most important information was relayed?*
  - *How did the receiver ensure the message received matched the message sent?*
  - *What suggestions would you give for improving this communication?*
- Refer all students to the questions listed following the case under the categories of: mutual respect, roles, communication and teams. Discuss these as a whole group.
  - Suggestions of appropriate answers are provided in red. These are not meant to be inclusive of all possible correct responses.
- Repeat with other three cases.

Cases

Pass out one of the scenarios to each of the four groups, in addition to the SBAR and Check-Back tables. Student are to discuss the case and complete the tables as described above.

Either use your own cases or one from available on-line resources. We used Scenarios from Jefferson InterProfessional Education Center Microsoft Word - SBAR IPE teaching plan.doc (jefferson.edu)

Scenario 1: Bill Jones

The Case:

Seventy-six year old admitted 3 days ago for sudden worsening of his chronic heart failure (heart is not pumping effectively and now he is short of breath). Yesterday, he was transferred from the Cardiac Intensive Care Unit (CICU) to a regular floor unit. This is your first day caring for Mr. Jones as a member of his healthcare team. He mentions to you how grateful he is for the eggs and bacon breakfast this morning and the tasty meal last night after the transfer to this hospital ward.

He said that the bland food in CICU was awful. You notice that his AM weight is up 2 pounds from his CICU discharge weight. You review his dietary order and it says "regular diet" which means no salt or fat restrictions. He has increased edema (swelling due to excess fluid) in his feet and his blood pressure is slightly elevated this morning (164/102) from last night's pressure of 152/98.

In your small group:

1) Discuss how you will use SBAR to convey this information. Fill in the SBAR table with your ideas.

2) Discuss how you will use the Check-Back guide to assure your understanding of the communicated information. Fill in the Check-Back table with your ideas.

3) Review and discuss the questions under the 4 categories below in preparation for the whole group discussion:

Mutual respect and shared values

- What questions would you ask the patient about his food choices? What aspects about his life might you consider?
  - Cultural diversity of food choices.

Roles

- What other healthcare professionals can assist with correct diet choices?
- What is the role of the electronic health record (EHR) for professionals in the care of this patient?
- What components of this case are relevant to the social environment and/or advocacy? At a Town Hall meeting with your congressional representative, what suggestions in health policy would you offer based on your experience?
  - Inclusion of health educator, social worker, and dieticians in the discharge planning.
  - EHR may play a role in developing order sets that assist with proper meal planning.
  - Assess healthy food options in the community, affordable medications, primary care.

Communication

- To whom and what should be communicated about dietary choices?
  - Communication should involve patient and families that might be making food choices.
  - Consider the entire family as part of the healthcare team.
  - Consider availability and access (i.e. cost) to healthy food sources within patient's/family's living environment.

Teams

- What team approaches could you consider to manage the medical error made in this case?
- Who is part of the team for development of his individual care plan and the well-being of the community health?
  - Team debrief to discuss the implications of the wrong diet.
  - Review of transfer process with healthcare and administrative team.
  - Effective health records that provide appropriate order sets.

Scenario 2: Joey Black

The Case:

Six-year-old male with a history of asthma. He is in the office for a well- child visit, and it is determined that his asthma has not been well controlled. He has episodes of wheezing on most days, which limits his physical activity; he has no other illnesses. His mother has expressed some confusion about when to use his rescue medications and how to best administer them since they must be inhaled. She is particularly concerned because her sister and mother both have terrible asthma, and she has seen what they went through. Joey had a flu shot this year, but mother has questions about why he needs so many vaccines. Joey is also scheduled for a tonsillectomy in a week.

In your small group:

1) Discuss how you will use SBAR to convey this information. Fill in the SBAR table with your ideas.

2) Discuss how you will use the Check-Back guide to assure your understanding of the communicated information. Fill in the Check-Back table with your ideas.

3) Review and discuss the questions under the 4 categories below in preparation for the whole group discussion:

Mutual respect and shared values

- His mother said she does not want to vaccinate Joey next fall because she read on the web that it may cause him to become autistic. How do you deal with this concern?

Roles

- What other roles may be involved in the care of Joey and education of his mother?
- The mother, who is a smoker, reports that their apartment is damp and moldy.
- As a member of the local health department advisory board, what actions would you recommend that they take to prevent episodes of asthma in the community?
  - Involve nurse, health educator, pharmacist, and social services if medication cost or living conditions are issues. Anaesthiology should be consulted prior to surgery. Mother may benefit from discussion with a genetic counselor about her family history.
  - Social worker may be of assistance. Primary care and smoking cessation information for the mother with an explanation of the effects of second-hand smoke on Joey.

- Local health department may help with community wide educational programs or advocate for better living conditions.

Communication

- What are some potential barriers to appropriate healthcare in this case and how can they best be communicated with the mother?
- You have learned that her apartment complex is damp and infested with cockroaches. How would you assist?
  - Actively listening and encouraging ideas.
  - Communication with local health departments to investigate.

Teams

- The office consists of a physician, an advanced practice provider a nurse, and a health educator. How should they work to deliver care?
- If you are presenting to the local health department advisory board recommendations to decrease childhood asthma in your community who would you want as part of your team?
  - Need to create plans on the best way to deliver care and education to both the patient and families. Clear care plans, educational material, scheduled educational appointment, regular huddles and more can help with delivering superior patient centered care.
  - Consider a team composed of both healthcare and public health experts to explain the medical care required, the impact on individual patients, community needs and impact on the community.

Scenario 3: Joan Willis

The Case:

Sixty-four-year-old female was brought to the hospital after "falling at home." Ms. Willis sustained an arm fracture, multiple bruises and a head laceration (cut). Over the telephone, you learn from her son that she lives alone and he has been concerned about her safety due to several falls. During those incidents she was evaluated at a local hospital and discharged home without contacting her son. The son lives several hours away and is driving to the hospital. She had a brief loss of consciousness after the fall. She was admitted to the observation unit overnight. Her orders include hourly neurovascular checks to track her mental status. Several hours into her observation unit stay her son brought in her medication list from home. One of her daily medications is warfarin, an anti-coagulant or blood thinner. During her last neurovascular check, it was noted that Ms. Willis had become somewhat confused, which was a change from the previous hour's assessment.

In your small group:

1) Discuss how you will use SBAR to convey this information. Fill in the SBAR table with your ideas.

2) Discuss how you will use the Check-Back guide to assure your understanding of the communicated information. Fill in the Check-Back table with your ideas.

3) Review and discuss the questions under the 4 categories below in preparation for the whole group discussion:

Mutual respect and shared values

- Assume the observation unit was at capacity. Should you reconsider your decision and discharge the patient home instead?
- What aspects of her presentation should you consider in your decision making?
- In addition, you just received an email to decrease admissions due to hospital overcrowding.
  - ➢ There are the medical concerns of the head injury. However, you also need to put the patient's interest at the center, development of trust with the son, incorporating the other concerns in your decision-making.

Roles

- Who else should you involve in her care and why?
- What are the needs of our aging population and how can we promote the health of this population?
  - ➢ Social work to assess home situation and assist the family. EHR experts to have quicker access to medications that could affect patient care. Home health care assessment by nurse and/or social worker to determine safety at home and whether you should stop the anticoagulation medication. Work with pharmacist on different options.
  - ➢ Our population is aging. Working with public health and advocacy groups to promote better in-home care and support would be of value to the individual patient and the community.

Communication

- You are the nurse for Ms. Willis and you want to call her primary care provider (PCP) to report her recent change in neurovascular status along with other pertinent information. You feel the change in her assessment may be due to a possible intracranial hemorrhage due to the fall and her use of blood thinners.
- How would you relay your concerns and desire to contact the PCP to the health care team?

- Who else may need to be contacted, and who should be involved?
    - SBAR can be used to relay the key information to the team. Their input can be sought on what needs to be communicated to the PCP.
    - Need to communicate change in status to the family first, so they do not receive an unexpected call from the PCP.

Teams

- What team exercises could help manage future patients similar to our patient today?
- How can the hospital personnel interact more effectively with the community to decrease these incidents?
    - Planning meetings to discuss care plans for head injury, admission criteria discussion, protocol driven orders for various hospital units, and more.
    - Could work to advocate for safer living conditions, better education of healthcare providers on the medical and social needs of living independently.

Scenario 4: Mary Smith

The Case:

Thirty-six year old healthy female patient with in utero diagnosis of trisomy 13, a chromosomal abnormality that typically leads to death in the first few days, weeks or months of life. There are however, longer-term survivors and groups that advocate for offering full surgical and medical intervention to prolong life. The condition is associated with multiple birth defects and severe to profound intellectual disability. Mary has been admitted in early labor at 30 weeks gestation. This is her first pregnancy; she and her husband have been actively involved in counseling with the decision to not prolong life with intensive medical care and heroic intervention. Their desire is to hold the baby and have what they term "good quality of life and death with dignity."

In your small group:

1) Discuss how you will use SBAR to convey this information. Fill in the SBAR table with your ideas.

2) Discuss how you will use the Check-Back guide to assure your understanding of the communicated information. Fill in the Check-Back table with your ideas.

3) Review and discuss the questions under the 4 categories below in preparation for the whole group discussion:

Mutual respect and shared values

- You are the nurse caring for Mary and feel strongly that this is not a sound ethical decision. What are some of your considerations before you enter the room to talk to the patient?
    - ➢ It is important to put the patient at the center of the care you provide, respect them and their decision, and develop trust.

Roles

- Who might be involved in the decision making process and during the hospital admission?
    - ➢ Besides the traditional clinical healthcare providers, consider Genetic Counselors, chaplaincy, mental health counselors.

Communication

- You are the charge nurse and have just learned their wishes. How do you communicate that to the rest of the care team?
    - ➢ Consider meeting with the team in advance (huddle) to discuss the decision and how the team will manage the care and feelings of all those involved.

Teams

- As the charge nurse you find one of your nurses crying about the situation. Around the corner you over hear a conversation by a tech about how they vehemently disagreed with the care plan. How do you manage this as the charge nurse?
- The husband is angry. While holding their child someone from environmental services came in to clean the room for the next patient. What could be done to prevent this intrusion?
    - ➢ An emotional case affects all members of the healthcare team differently. A debrief session run by someone with expertise in debriefing emotionally charged situations will provide an opportunity for open discussion and reflection.
    - ➢ Establishment of a care delivery plan involving all aspects of inpatient care would help decrease the chance of this type of mistake. Involvement of the entire team would allow for everyone to be on a shared model of care and provide for better communication.
